# Changes in Circulation and Particle Scavenging in the Amerasian Basin of the Arctic Ocean over the Last Three Decades Inferred from the Water Column Distribution of Geochemical Tracers

**DOI:** 10.1029/2019JC015265

**Published:** 2019-12-18

**Authors:** Melanie Grenier, Roger François, Maureen Soon, Michiel Rutgers van der Loeff, Xiaoxin Yu, Ole Valk, Christelle Not, S. Bradley Moran, R. Lawrence Edwards, Yanbin Lu, Kate Lepore, Susan E. Allen

**Affiliations:** ^1^ Department of Earth, Ocean and Atmospheric Sciences University of British Columbia Vancouver British Columbia Canada; ^2^ Alfred Wegener Institute, Helmholtz Centre for Polar and Marine Research Bremerhaven Germany; ^3^ Department of Earth Sciences The University of Hong Kong Hong Kong; ^4^ College of Fisheries and Ocean Sciences University of Alaska Fairbanks Fairbanks AK USA; ^5^ Department of Earth Sciences University of Minnesota Minneapolis MN USA; ^6^ Mount Holyoke College South Hadley MA USA

**Keywords:** Arctic Ocean, Amerasian Basin, radioisotopes, temporal evolution, particle flux, lateral exchanges

## Abstract

Since the 1980–1990s, international research efforts have augmented our knowledge of the physical and chemical properties of the Arctic Ocean water masses, and recent studies have documented changes. Understanding the processes responsible for these changes is necessary to be able to forecast the local and global consequences of these property evolutions on climate. The present work investigates the distributions of geochemical tracers of particle fluxes and circulation in the Amerasian Basin and their temporal evolution over the last three decades (from stations visited between 1983 and 2015). Profiles of 230‐thorium (^230^Th) and 231‐protactinium (^231^Pa) concentrations and neodymium isotopes (expressed as ε_Nd_) measured in the Amerasian Basin prior to 2000 are compared to a new, post‐2000s data set. The comparison shows a large scale decrease in dissolved ^230^Th and ^231^Pa concentrations, suggesting intensification of scavenging by particle flux, especially in coastal areas. Higher productivity and sediment resuspension from the shelves appear responsible for the concentration decrease along the margins. In the basin interior, increased lateral exchanges with the boundary circulation also contribute to the decrease in concentration. This study illustrates how dissolved ^230^Th and ^231^Pa, with ε_Nd_ support, can provide unique insights not only into changes in particle flux but also into the evolution of ocean circulation and mixing.

## Key Points

- Concentration decreases of ^230^Th_d_ and ^231^Pa_d_ suggest an enhancement of particulate scavenging since the 2000s in the Amerasian Basin
- Particulate scavenging results from higher productivity and sediment resuspension from the shelves
- Post‐2000s changes in intermediate and deep layers suggest enhanced lateral mixing between the margins and central areas of the basins

## 1 Introduction

**Figure 1:**
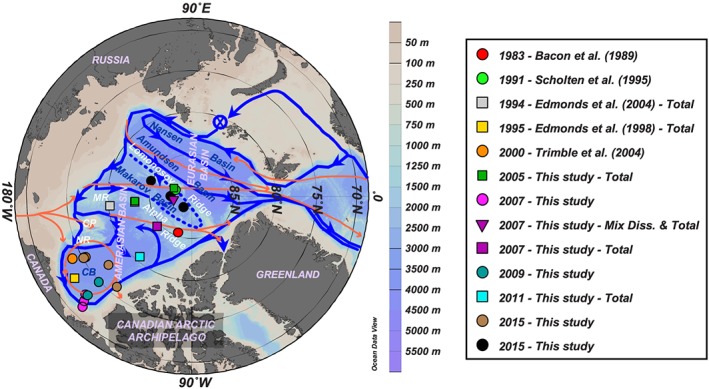
Location of the deep stations (>500 m depth) where ^230^Th, ^231^Pa, or ε_Nd_ were measured in the Amerasian Basin of the Arctic so far. This study adds nine new stations in the Canada Basin (CB; 2007: pink dots; 2009: blue‐green dots; 2011: cyan square; 2015: brown dots), one new on the Mendeleev Ridge (MR; 2005: green square), five in the Makarov Basin (2005: green square; 2007: purple inverted triangle; 2015: black dots), and one on the Alpha Ridge (2007: purple square). The red arrows show the circulation schematics of the upper layers; the blue ⊗ and arrows show the formation area and circulation schematics of the Atlantic layer, respectively (after Bluhm et al., 2015; Rudels et al., 2013). CP = Chukchi Plateau; NR = Northwind Ridge. This map and the following were created with the software Ocean Data View (Schlitzer, 2015), using the IBCAO bathymetry (version 3; Jakobsson et al., 2012).

The pathways that waters follow in the ocean and the physicochemical changes they undergo along these pathways dictate the redistribution of key parameters such as heat, salt, energy, nutrients, and pollutants. It is of particular interest to examine the pathways and fate of waters in the Arctic Ocean because some of these waters eventually exit to enter the Atlantic Ocean and impact North Atlantic deep water formation and the global overturning circulation (Aagaard et al., 1985; Hansen & Østerhus, 2000). Arctic Ocean waters are made of Pacific and Atlantic Ocean waters that enter the Amerasian Basin through the shallow Bering Strait and the Eurasian Basin through the shallow Barents Sea and deep Fram Strait. They eventually return to the Atlantic Ocean through Fram Strait and the relatively shallow Canadian Arctic Archipelago as upper or intermediate waters (Aagaard & Greisman, 1975). During their transit in the Arctic Ocean, these waters are transported by the upper wind‐driven circulation and the intermediate cyclonic topographical‐steered circulation (Figure 1; Rudels, 2001; Rudels et al., 2013).

Investigating the pathways and fate of waters in the Arctic Ocean is all the more important as the Arctic Ocean represents one of the most rapidly changing regions of the world's oceans (Pörtner et al., 2015). With a reduction in seasonal sea ice coverage, changes in hydrography and water mass circulation have been observed, not only at the surface but also at depth (e.g., Polyakov et al., 2013). Particle concentrations are also projected to increase in the rapidly changing Arctic Ocean, as a result of increased continental runoff and biological production, impacting the chemical properties of Arctic waters and downstream (e.g., Carmack et al., 2006).

The first observed changes in temperature—abnormally warm Atlantic water (hereafter, awAW)—occurred in 1990 in Fram Strait (Quadfasel et al., 1991). This awAW propagated through the whole Arctic, first into the Eurasian Basin, then beyond the Lomonosov Ridge, and into the Amerasian Basin. This temperature anomaly was observed in the southern Makarov Basin (MB) and Mendeleyev Ridge in 1993 (Carmack et al., 1995), in the central MB in 2000 (Kikuchi et al., 2005), at the northern tip of the Northwind Ridge in 2003 (Woodgate et al., 2007), and within most of the Canada Basin (CB) in 2007 (McLaughlin et al., 2009; see Figure 1 for the basin and ridge locations). However, these studies did not reveal significant changes in circulation related to the awAW propagation in the Arctic. Likewise, no significant changes in particle distribution were reported from limited measurements in the CB conducted between 2003 and 2008 (Jackson et al., 2010). However, the latter study documented a sharp contrast between low particle concentrations of the central basin and much higher concentrations near the CB continental margin.

Because of their affinity for particles and residence times similar to the time scale of regional changes occurring in the Arctic, geochemical tracers such as ^230^Th and ^231^Pa are powerful tools to quantify the recent evolution of particle flux and circulation in the Arctic Ocean. ^230^Th and ^231^Pa are produced in the ocean by radioactive α‐decay of uranium‐234 (^234^U) and uranium‐235 (^235^U), respectively; uranium input to the ocean occurs through continental weathering. Uranium is soluble in seawater and has a long residence time in the ocean (~400,000 years; Brewer, 1975; Chen et al., 1986), such that its concentration and the rate of production of ^230^Th and ^231^Pa are uniform and well known (Ku et al., 1977; Turekian & Chan, 1971). Unlike parent U, Th and Pa—especially Th—are highly insoluble in seawater and effectively removed to the sediment by adsorption onto sinking particles (Anderson et al., 1983), a process referred to as particulate scavenging. As a result, ^230^Th residence times in seawater range from a few years in shallow water to a few decades in deep water; for ^231^Pa, residence times range from a few decades in shallow water to a few centuries in deep water (Henderson & Anderson, 2003). If transport of ^230^Th and ^231^Pa by advection and turbulent diffusion can be neglected, the oceanic distribution of these radionuclides is largely controlled by reversible scavenging: dissolved radionuclides (R_d_; unit: concentration) are produced continuously at fixed rates (α; unit: concentration per time) and adsorbed reversibly onto sinking particles (*k*_*a*_ and *k*_*d*_ are adsorption and desorption coefficients; unit: per time) to produce sinking particulate radionuclides (R_*p*_; unit: concentration), removed at a sinking rate S (unit: length per time) to the underlying sediment (Bacon & Anderson, 1982; Nozaki et al., 1981). Thus, neglecting advection and diffusion, the conservation equations for the radionuclide dissolved and particulate concentrations ([R_*d*_] and [R_*p*_], respectively) are given by

(1) $$\frac{\partial {\left[R\right]}_{d}}{\mathrm{\partial t}}=\alpha +k_{d}\cdot {\left[R\right]}_{p}-k_{a}\cdot {\left[R\right]}_{d}$$

(2) $$\frac{\partial {\left[R\right]}_{p}}{\mathrm{\partial t}}=k_{a}\cdot {\left[R\right]}_{d}-k_{d}\cdot {\left[R\right]}_{p}-S\cdot \frac{\partial {\left[R\right]}_{p}}{\mathrm{\partial z}}$$

where *z* is depth, increasing downward. The sinking flux is the last term in equation (2). Under assumptions of steady state and constant S, *k*_*a*_ and *k*_*d*_, equations (1) and (2) predict a linear increase of ^230^Th and ^231^Pa concentrations with depth, with a slope inversely proportional to the sinking rate of particles:

(3) $${\left[R\right]}_{d}=\frac{\alpha }{k_{a}}+\frac{k_{d}\cdot \alpha }{k_{a}\cdot S\;}\cdot z$$

(4) $${\left[R\right]}_{p}=\frac{\;\alpha }{\;S\;}\cdot z$$

**Figure 2:**
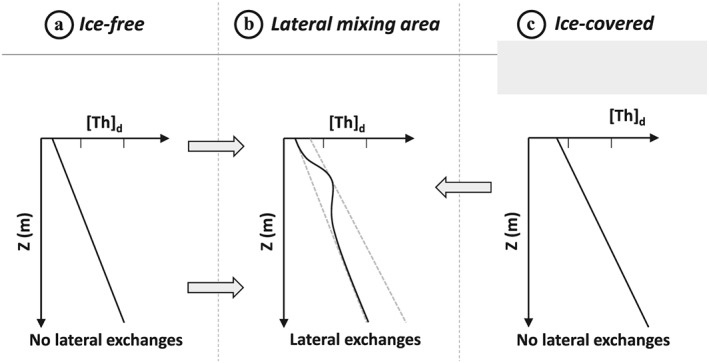
Schematic representation of dissolved ^230^Th concentration profiles (a, c) following equation (3), that is, neglecting advection and turbulent diffusion and assuming steady state, in (a) a seasonally ice‐free area, where the surface value is lower and the increase with depth is weaker due to higher scavenging and particulate sinking rates than in (c) a permanently ice‐covered area. Profiles with deviations from linearity are expected in (b) areas where lateral advection of ^230^Th from (a) (low grey dotted line) and (c) (high grey dotted line) occurs (grey arrows).

Based on observations of a strong positive correlation between *k*_*a*_ and the concentration of suspended matter (Bacon & Anderson, 1982), we expect to find lower concentrations and a reduced downward increase of dissolved concentrations in areas of high particle concentrations and flux. For example, this scenario is expected for the seasonally ice‐free CB continental margin (Figure 2a), compared to higher concentrations and a faster downward increase in areas of low particle concentrations and flux, such as the permanently ice‐covered central basin (Figure 2c).

These simplified schematics actually reproduce quite well the ^230^Th and ^231^Pa oceanic distributions of the Amerasian Basin reported from the first observations, in the 1980–1990s. Low and linear vertical profiles were observed in 1995 in the southern CB, reflecting the integrated impact of particle scavenging in the boundary currents (Edmonds et al., 1998). In contrast, higher concentrations were found over the Alpha Ridge in 1983 and in the northern MB in 1991 (Bacon et al., 1989; Scholten et al., 1995; see Figure 1 for station locations). Such high concentrations of ^230^Th and ^231^Pa in seawater *not only* suggest that particle concentrations and fluxes were locally very low *but also* that these waters had been isolated for a few decades from the dynamic boundary circulation, allowing for in‐growth of ^230^Th and ^231^Pa. A subsequent increase in particle flux should lead to a decrease in dissolved ^230^Th and ^231^Pa concentrations. Furthermore, as the affinity of ^230^Th and ^231^Pa changes with their particle composition (e.g., Chase et al., 2002), differences in the evolution of the distribution of ^230^Th relative to ^231^Pa (i.e., decoupling) could also occur depending on particle composition (e.g., biogenic silica vs. lithogenic particles). In addition, changes in horizontal mixing or advection rates between the basin interior and the margins could decrease the contrast in ^230^Th and ^231^Pa concentrations between the two regions or even produce subsurface maxima in the concentration profiles (see Figure 2b). Note that the assumption of constant S and *k*_*a*_ relies on a constant vertical flux of particulate ^230^Th, which is a good approximation as organic matter decomposition does not release significant amounts of ^230^Th—or ^231^Pa—to seawater because of the high particle reactivity of these radionuclides.

The present work aims to investigate changes in particle scavenging and circulation over the last three decades in the Amerasian Basin by comparing ^230^Th and ^231^Pa concentration profiles collected in the 1980s and 1990s to 13 water‐column profiles collected between 2005 and 2015. A companion paper (Yu et al., n.d.) further exploits these data by introducing ^230^Th scavenging into a three‐dimensional model hindcasting Arctic circulation and particle scavenging between 2002 and 2015.

We also report three dissolved profiles of neodymium isotopic compositions from 2015 that are compared to early‐2000s data (Porcelli et al., 2009) and support the interpretation of the ^230^Th‐^231^Pa profiles. Neodymium (Nd) is a rare earth element (REE) that is supplied to the ocean via continental weathering. As the neodymium isotopic composition (expressed as ε_Nd_) of rocks varies with age and lithology, the ε_Nd_ of outcropping rocks is heterogeneous (Jeandel et al., 2007). Thus, the ε_Nd_ signal of the water masses is set by the continental Nd they receive along their circulation pathways (through direct input or through mixing with a water mass of different ε_Nd_; e.g., Frank, 2002; Goldstein & Hemming, 2003; Grenier et al., 2013; Piepgras et al., 1979; Tachikawa et al., 2003). Pacific waters in the Bering Strait have ε_Nd_ values of ~−5 (Dahlqvist et al., 2007), while Atlantic waters have values of ~−11 (Andersson et al., 2008). These distinct signatures allowed Porcelli et al. (2009) to trace distinct water masses and follow the evolution of their ε_Nd_ during their transit in the Arctic in the early‐2000s.

## 2 Water masses of the Amerasian Basin

**Figure 3:**
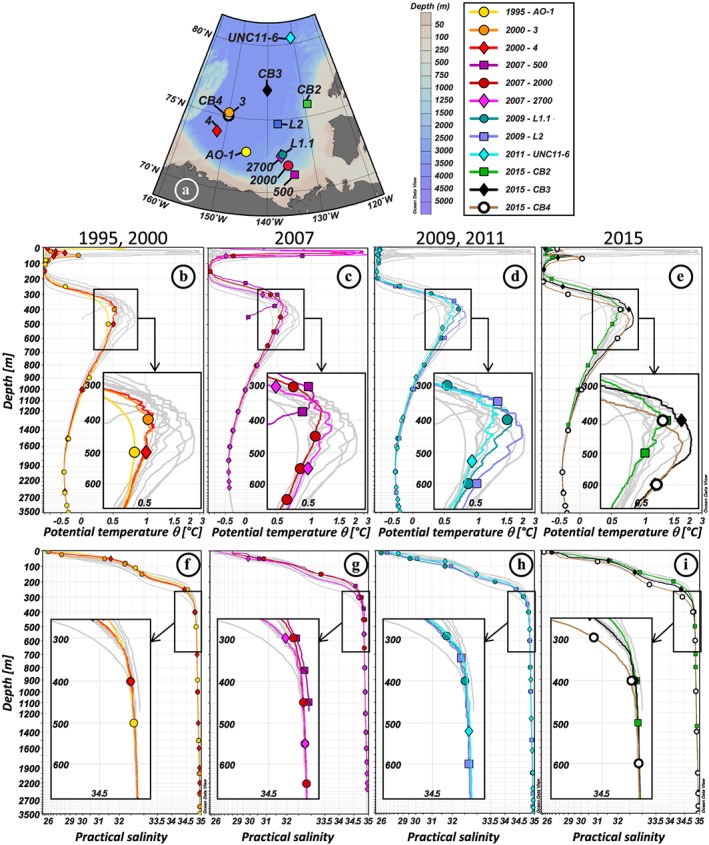
Vertical profiles of hydrological parameters of the Canada Basin stations. (a) Location of the stations. Vertical profiles of potential temperature (°C; b–e) and practical salinity (f–i) of the 12 stations, in grey, superimposed by the colored profiles of stations sampled in 1995 and 2000 (b, f), 2007 (c, g), 2009 and 2011 (d, h), and 2015 (e, i). Hydrological references: Carmack et al. (1996) (1995: AO‐1); Porcelli et al. (2009) (2000: 3, 4); McLaughlin et al. (2009) (2007: 500, 2000, 2700); Rail et al. (2011) (2009: L1.1, L2); Mosher (2012) (2011: UNC11‐6); Amundsen Science Data Collection (2016) (2015: CB2, CB3, CB4). Symbols on the colored profiles represent the samples collected at each station. For the sake of clarity, horizontal and vertical axes are stretched in order to better visualize the properties of the Atlantic layer, and a zoom on this layer, defined by the black square, is shown in an inset for each plot.

**Figure 4:**
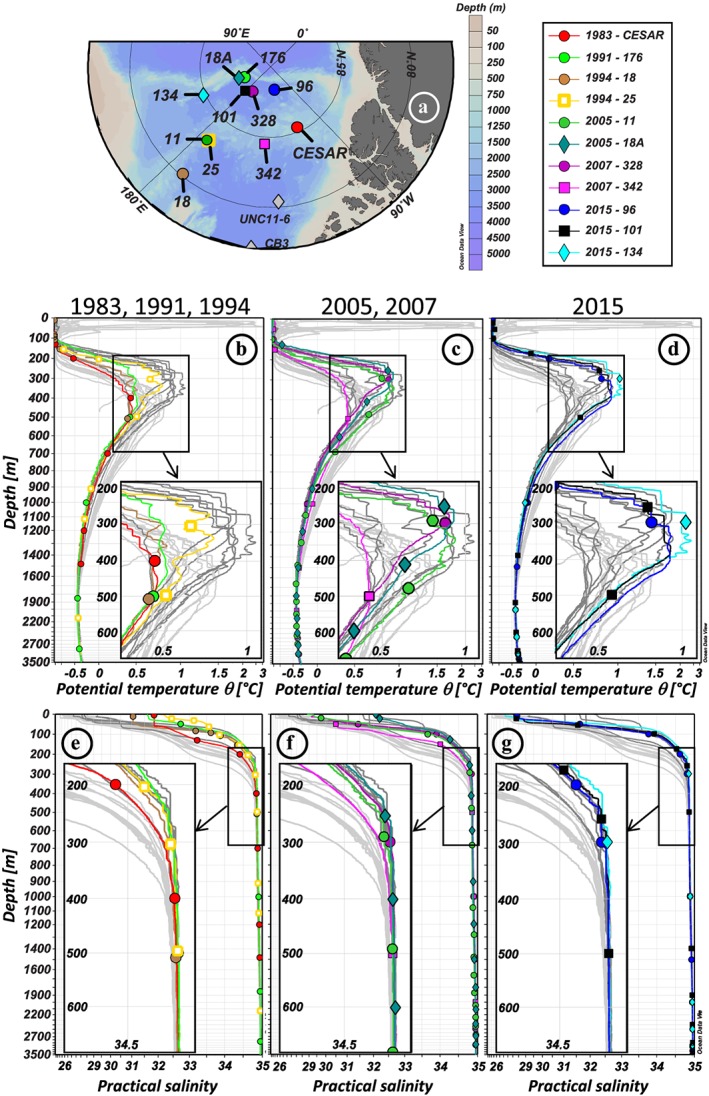
Vertical profiles of hydrological parameters of the Makarov Basin and ridges stations. (a) Location of the stations. Vertical profiles of potential temperature (°C; b–d) and practical salinity (e–g) of the 11 stations, in dark grey, superimposed by the colored profiles of stations sampled in 1983, 1991, and 1994 (b, e), 2005 and 2007 (c, f), and 2015 (d, g). Canada Basin light grey profiles are also reported in the background, for comparison. Hydrological references: Jones and Anderson (1986) (1983: CESAR); Anderson et al. (1994) (1991: 176); Swift et al. (1997) (1994: 18, 25); Darby et al. (2006) (2005: 11, 18A); Schauer (2008) (2007: 328, 342); Rabe et al. (2016) (2015: 96, 101, 134). Symbols on the colored profiles represent the samples collected at each station. For the sake of clarity, horizontal and vertical axes are stretched in order to better visualize the properties of the Atlantic layer, and a zoom on this layer, defined by the black square, is shown in an inset for each plot.

The water column in the Amerasian Basin can be separated vertically into three layers, recognizable in Figures 3 and 4 and supporting information Figure S1: (i) the low‐salinity Polar Surface Water (PSW) including the Polar Mixed Layer (PML) and the halocline; (ii) the warm Atlantic Water, identified as a subsurface layer with a temperature maximum bounded above and below by the 0 °C isotherm, and the underlying intermediate water; and (iii) colder and more saline deep and bottom waters. The θ‐S profiles presented in Figure S1 are subdivided following these three layers.

The PML is the homogenized surface layer undergoing brine rejection and haline convection in winter and receiving fresh water from sea ice melt in summer. The underlying halocline water originates from the Pacific Ocean and includes the fresher and warmer Alaskan Coastal Water (ACW) overlying the saltier and colder Bering Sea Water (BSW; Coachman & Barnes, 1961; Steele et al., 2004; Timmermans et al., 2014). The ACW is characterized by a subsurface temperature maximum in the salinity range [29–32.2], usually found between 50 and 100 m depth. The BSW has a summer and winter component. The Bering Sea Winter Water (BSWW) is more easily distinguished than the Bering Sea Summer Water (BSSW) and more widely spread. The denser BSWW is identified by a temperature minimum (~−1.6 °C) at S ~33 while the BSSW can be found in the salinity range [32.2–33] and is characterized, like the overlying ACW, by a temperature maximum. The halocline extends to gradually shallower depths from the CB (200–250 m), to the Alpha Ridge (150–200 m), and to the MB (100–150 m). Below lies the Atlantic layer, marked by a prominent temperature maximum. This water is mostly derived from the Barents Sea branch (Rudels et al., 2013) and found between 250 and 1,000 m depth in the CB, between 250 and 800/900 m depth on the Alpha Ridge and between 200 and 800 m depth in the MB. Deeper, bounded by the 0 °C temperature above and by the deep temperature minimum at about 2,400 m depth is the upper Polar Deep Water (uPDW; Rudels, 2009). Finally, below the 2,400 m temperature minimum, which marks the depth of lateral exchange between the Canada and Makarov Basins (E. Carmack et al., 2012), are the Canada Basin Deep Water (CBDW) and the Makarov Basin Deep Water (MBDW). The higher salinity of the CBDW and the MBDW compared to the Eurasian Basin bottom water could be explained by their relative isolation and the formation of dense water by brine rejection on the shelves (Aagaard et al., 1985; Jones et al., 1995; Mauritzen et al., 2013). CBDW temperature is also slightly higher, suggesting geothermal heating and mixing (E. Carmack et al., 2012), while MBDW gradually cools toward the bottom. There may be some exchange of deep water with the Eurasian Basin where the Lomonosov Ridge deepens to 1,800 m (close to the North Pole, around 175°E; Cochran et al., 2006), but the flow appears to be mostly from the MB to the Amundsen Basin (Rudels, 2009). While surface and halocline water circulation is anticyclonic in the CB and driven by the Transpolar Drift above the Lomonosov Ridge, the Atlantic layer below mainly follows topographically steered cyclonic gyres (circulation schematics in Figure 1). The circulation of bottom water remains virtually unknown. The different water masses described here have been sampled at most of the stations reported in this study (samples are identified by markers in Figures 3, 4, and S1).

## 3 Methods

New ^230^Th, ^231^Pa, and ε_Nd_ data presented in this study (Tables 1 and 2) are from ~15 L seawater samples collected during seven different cruises (Figure 1). Four cruises were conducted in the CB: (1) in September 2007, on the CCGS Sir Wilfrid Laurier; (2) in September 2009, on the CCGS Amundsen (GEOTRACES section IPY14); (3) in September 2011, on the CCGS Louis St‐Laurent; and (4) in September 2015, on the CCGS Amundsen (GEOTRACES section GN03). The fifth cruise, a Trans Arctic Expedition (HOTRAX), occurred in August–September 2005, aboard the USCG Healy. The sixth and seventh cruises were mostly conducted in the Eurasian Basin, on‐board the R/V Polarstern, in 2007 (GEOTRACES section IPY11) and 2015 (GEOTRACES section GN04; Schauer, 2016), respectively. In our study, we only report five stations from these last two cruises: two collected in the MB and Alpha Ridge area in September 2007 and three in the MB in September 2015. Eurasian Basin ^230^Th profiles from these two cruises are reported in Valk et al. (2018).

**Table 1:** Dissolved ^230^Th and ^231^Pa concentrations (in fg kg^−1^), and neodymium isotopic compositions (expressed as ε_Nd_, unitless) of the Canada Basin stations collected in 2015, with their associated standard errors and hydrological properties (potential temperature, in °C; salinity, unitless; potential density, in kg m^−3^)

Depth (m)	θ (°C)	Salinity	σ_θ_ (kg m^−3^)	^230^Th (fg kg^−1^)	SE (95%)	^231^Pa (fg kg^−1^)	SE (95%)	ε_Nd_	SE (95%)
**Canada** **Basin**									
CB2 (9 September 2015; −129.234°E, 75.806°N; depth: 1350 m)									
10	−0.677	27.058	21.716	1.89	0.07	0.05	0.07	−8.0	0.1
65	−0.773	31.661	25.441	2.03	0.07	0.29	0.19	−5.8	0.1
140	−1.399	32.895	26.459	1.33	0.04	0.17	0.07	−5.8	0.1
200	−0.819	34.112	27.426	2.05	0.20	0.17	0.09	−8.1	0.1
400	0.598	34.821	27.926	4.20	0.11	0.54	0.05	−9.9	0.2
400	0.598	34.821	27.926	—	—	0.54	0.06	−10.3	0.2
500	0.465	34.842	27.951	3.54	0.10	0.90	0.09	−9.7	0.2
700	0.205	34.863	27.983	2.98	0.10	0.54	0.07	−9.3	0.2
800	0.100	34.870	27.994	2.97	0.09	0.58	0.07	−9.3	0.2
1,000	−0.083	34.882	28.013	3.22	0.09	0.46	0.07	−9.4	0.1
1,338	—	—	—	1.98	0.07	0.44	0.07	−9.4	0.1
CB3 (12 September 2015; −140.053°E, 76.975°N; depth: 3,730 m)									
10	−1.284	26.539	21.303	1.75	0.05	0.18	0.05	−8.2	0.2
65	−0.399	31.443	25.251	1.97	0.06	*<DL*	—	−7.1	0.1
140	−1.375	32.679	26.283	1.15	0.06	*<DL*	—	−6.3	0.1
250	−0.455	34.348	27.602	2.92	0.07	—	—	−8.4	0.1
400	0.693	34.795	27.899	5.66	0.11	0.66	0.04	−10.4	0.1
600	0.539	34.848	27.951	6.57	0.12	0.68	0.06	−9.9	0.3
1,001	−0.050	34.874	28.005	8.66	0.13	0.96	0.07	−10.3	0.2
1,400	−0.318	34.908	28.049	11.15	0.24	1.34	0.11	−10.1	0.2
1,400	−0.318	34.908	28.049	10.93	0.37	1.42	0.09	−10.5	0.3
2,001	−0.509	34.942	28.085	10.46	0.23	2.11	0.11	−9.9	0.2
2,459	−0.519	34.953	28.094	12.31	0.23	3.11	0.12	−9.8	0.2
3,000	−0.508	34.957	28.097	11.92	0.26	2.33	0.16	−9.7	0.1
3,496	−0.507	34.957	28.097	12.65	0.32	2.36	0.11	−9.9	0.2
CB4 (15 September 2015; −150.001°E, 75.002°N; depth: 3,830 m)									
10	−0.479	25.27	20.268	2.22	0.05	0.29	0.04	−8.2	0.1
71	0.047	31.014	24.887	2.39	0.07	0.05	0.06	−9.0	0.2
220	−1.476	33.248	26.748	1.81	0.06	*<DL*	—	−6.3	0.1
300	−0.265	34.424	27.654	2.56	0.09	0.06	0.06	−8.1	0.1
400	0.609	34.775	27.889	4.30	0.11	0.82	0.06	−10.4	0.3
600	0.564	34.843	27.945	2.34	0.08	0.80	0.06	−8.9	0.2
1,000	−0.025	34.870	28.000	3.49	0.09	0.98	0.08	−10.1	0.1
1,400	−0.320	34.902	28.044	6.32	0.15	1.27	0.05	−10.3	0.1
1,400	−0.320	34.902	28.044	6.56	0.21	1.34	0.09	−10.4	0.2
2,000	−0.504	34.940	28.084	10.21	0.22	1.87	0.06	−9.5	0.1
3,000	−0.507	34.957	28.097	11.57	0.24	3.13	0.11	−9.6	0.1
3,500	−0.507	34.957	28.097	11.62	0.26	2.29	0.09	−10.2	0.5

**Table 2:** Dissolved (or total, in italic) ^230^Th and ^231^Pa concentrations (in fg kg^−1^) of the Mendeleev Ridge, Makarov Basin, and Alpha Ridge stations, collected in 2005, 2007, and 2015, with their associated standard errors and hydrological properties (potential temperature, in °C; salinity, unitless; potential density, in kg m^−3^)

Depth (m)	θ (°C)	Salinity	σ_θ_ (kg m^−3^)	^230^Th (fg kg^−1^)	SE (95%)	^231^Pa (fg kg^−1^)	SE (95%)
Canada Basin							
500 (1 October 2007; −134.364°E, 71.077°N; depth: 500 m)							
50	0.798	30.550	24.484	0.71	0.08	0.33	0.07
150	−1.377	33.345	26.832	0.87	0.09	0.11	0.09
225	−0.030	34.559	27.756	0.79	0.12	0.05	0.07
300	0.488	34.766	27.893	0.44	0.06	0.20	0.05
375	0.445	34.841	27.957	0.63	0.11	0.20	0.04
375	0.444	34.841	27.957	0.74	0.08	0.20	0.07
450	0.021	34.881	28.013	1.01	0.10	0.37	0.10
450	0.020	34.881	28.013	1.19	0.11	0.37	0.07
2000 (30 September 2007; −135.497°E, 71.732°N; depth: 2,000 m)							
50	−0.073	30.816	24.738	1.20	0.14	—	—
150	−1.399	33.357	26.842	1.06	0.10	—	—
300	0.389	34.737	27.876	1.77	0.15	0.19	0.07
450	0.526	34.829	27.942	1.47	0.09	0.10	0.07
550	0.424	34.847	27.962	1.73	0.12	0.41	0.07
650	0.327	34.859	27.977	1.83	0.12	0.31	0.07
800	0.141	34.871	27.998	1.95	0.13	0.35	0.06
1,000	−0.063	34.886	28.020	2.49	0.13	0.35	0.11
1,200	−0.221	34.899	28.040	3.27	0.20	0.46	0.08
1,400	−0.356	34.914	28.059	2.32	0.16	0.45	0.07
1,600	−0.448	34.928	28.075	2.47	0.17	1.19	0.12
1,800	−0.491	34.942	28.089	2.31	0.17	1.17	0.09
2700 (29 September 2007; −136.936°E, 72.415°N; depth:2,700 m)							
50	−0.365	29.831	23.954	2.01	0.31	0.13	0.18
300	0.269	34.661	27.822	4.44	0.22	0.19	0.17
550	0.473	34.845	27.958	6.34	0.39	0.34	0.16
800	0.161	34.870	27.996	6.32	0.34	0.50	0.11
1,000	−0.056	34.885	28.019	5.65	0.29	1.07	0.17
1,200	−0.218	34.898	28.038	4.98	0.26	0.61	0.13
1,400	−0.343	34.911	28.056	3.81	0.21	1.21	0.17
1,600	−0.438	34.924	28.072	2.82	0.23	0.89	0.12
1,800	−0.489	34.937	28.084	3.73	0.14	2.07	0.23
2,000	−0.505	34.945	28.091	4.45	0.28	2.37	0.32
2,200	−0.511	34.950	28.096	4.30	0.22	2.43	0.15
2,349	−0.510	34.953	28.099	5.47	0.34	2.99	0.24
L1.1 (9 September 2009; −136.599°E, 72.514°N; depth: 2,530 m)							
9	−1.140	26.155	20.991	0.79	0.24	0.50	0.08
50	−1.321	29.650	23.825	1.79	0.29	1.05	0.08
100	−1.214	31.762	25.535	1.70	0.24	0.85	0.11
200	−1.294	33.697	27.107	2.90	0.22	0.74	0.09
250	−0.294	34.390	27.628	3.53	0.23	0.70	0.10
300	0.292	34.639	27.798	4.70	0.42	0.87	0.10
400	0.683	34.802	27.906	5.44	0.26	0.88	0.07
600	0.422	34.841	27.953	4.00	0.26	1.13	0.09
800	0.160	34.858	27.981	4.26	0.38	1.37	0.16
1,000	−0.059	34.873	28.005	3.68	0.32	1.03	0.06
1,500	−0.406	34.907	28.048	2.95	1.28	0.79	0.06
2,000	−0.506	34.934	28.074	3.97	0.26	1.63	0.11
L2 (4 September 2009; −137.383°E, 74.653°N; depth: 3,600 m)							
10	−1.392	26.576	21.334	0.21	0.19	0.32	0.03
50	−1.214	30.100	24.188	0.78	0.20	0.04	0.05
100	−1.173	31.974	25.706	1.06	0.22	0.54	0.05
250	−0.409	34.375	27.621	3.47	0.24	—	—
350	0.645	34.768	27.881	4.59	0.21	0.80	0.07
600	0.474	34.840	27.949	6.99	0.24	0.90	0.07
1,000	−0.029	34.873	28.003	9.98	0.27	1.25	0.07
1,500	−0.368	34.906	28.046	9.82	0.29	0.94	0.09
2,000	−0.511	34.930	28.071	8.97	0.44	3.13	0.13
2,500	−0.520	34.941	28.078	8.45	0.29	1.06	0.07
3,000	−0.509	34.945	28.079	9.66	0.58	2.54	0.15
UNC11‐6 (18 September 2011; −130.745°E, 80.37°N; depth: 3,480 m)							
20^a^	−1.160	29.135	23.406	*1.36*	*0.19*	—	—
70^a^	−1.151	31.724	25.503	*1.74*	*0.22*	—	—
160^a^	−1.501	33.063	26.598	*1.46*	*0.20*	*0.21*	*0.08*
525^a^	0.453	34.845	27.955	*9.69*	*0.26*	*0.71*	*0.05*
900^a^	0.015	34.879	28.008	*12.29*	*0.27*	*0.86*	*0.05*
1,265^a^	−0.271	34.905	28.043	*13.10*	*0.29*	*1.14*	*0.09*
1,630^a^	−0.443	34.927	28.069	*15.10*	*0.74*	*1.71*	*0.12*
2,000^a^	−0.512	34.943	28.086	*15.23*	*0.29*	*2.02*	*0.07*
2,365^a^	−0.520	34.951	28.093	*15.81*	*0.29*	*1.87*	*0.09*
2,735^a^	−0.510	34.955	28.096	*18.02*	*0.29*	*2.48*	*0.08*
3,100^a^	−0.508	34.956	28.096	*17.59*	*0.39*	*2.05*	*0.12*
3,470^a^	−0.508	34.956	28.096	*17.78*	*0.46*	*2.00*	*0.13*
Mendeleev Ridge							
11 (25 August 2005; −174.92°E, 83.11°N; depth: 2,700 m)							
20^a^	−1.551	29.845	23.987	*1.43*	*0.31*		
99^a^	−1.679	33.695	27.115	*2.64*	*0.44*		
290^a^	0.805	34.791	27.891	*5.57*	*0.52*		
485^a^	0.634	34.852	27.950	*8.37*	*0.56*		
683^a^	0.223	34.859	27.980	*7.39*	*0.45*		
1,078^a^	−0.240	34.883	28.024	*9.42*	*0.72*		
1,273^a^	−0.367	34.897	28.042	*9.71*	*0.36*		
1,470^a^	−0.429	34.914	28.059	*13.06*	*0.83*		
1,667^a^	−0.499	34.925	28.071	*12.01*	*0.53*		
1,862^a^	−0.498	34.939	28.083	*14.44*	*0.79*		
2,059^a^	−0.505	34.946	28.088	*15.68*	*0.54*		
2,254^a^	−0.506	34.949	28.091	*17.47*	*0.75*		
2,439^a^	−0.506	34.951	28.093	*18.14*	*0.92*		
2,646^a^	−0.505	34.954	28.095	*23.42*	*0.94*		
Alpha Ridge							
342 (7 September 2007; −138.413°E, 84.500°N; depth: 2,275 m)							
50^a^	−1.581	30.5079	24.526	*1.57*	*0.03*	*0.05*	*0.01*
150^a^	−1.376	34.0082	27.362	*5.14*	*0.06*	*0.08*	*0.01*
500^a^	0.396	34.8605	27.971	*15.11*	*0.14*	*1.23*	*0.04*
1,000^a^	−0.126	34.9013	28.033	*14.34*	*0.13*	*1.39*	*0.04*
1,001^a^	−0.127	34.9013	28.033	*14.48*	*0.13*	*1.47*	*0.04*
1,500^a^	−0.416	34.9282	28.070	*14.75*	*0.13*	*1.73*	*0.06*
2,200^a^	−0.503	34.952	28.093	*11.7* *0*	*0.11*	*2.42*	*0.05*
Makarov Basin							
18A (6 September 2005; 156.216°E, 87.628°N; depth: 4,000 m)							
2^a^	−1.737	32.069	25.796	*1.60*	*0.31*		
21^a^	−1.741	32.137	25.852	*1.90*	*0.43*		
126^a^	−0.858	34.263	27.551	*5.24*	*0.36*		
255^a^	0.894	34.810	27.900	*12.86*	*0.73*		
404^a^	0.660	34.855	27.951	*9.64*	*0.55*		
603^a^	0.290	34.868	27.984	*10.59*	*0.42*		
902^a^	−0.124	34.884	28.020	*12.72*	*0.68*		
1,201^a^	−0.324	34.907	28.048	*16.84*	*0.70*		
1,501^a^	−0.438	34.928	28.070	*20.78*	*0.90*		
1,802^a^	−0.489	34.942	28.084	*24.30*	*0.97*		
2,105^a^	−0.509	34.948	28.090	*27.72*	*1.25*		
2,401^a^	−0.516	34.951	28.093	*27.09*	*0.90*		
2,705^a^	−0.523	34.952	28.094	*27.61*	*0.90*		
3,008^a^	−0.530	34.952	28.094	*30.67*	*0.90*		
3,649^a^	−0.534	34.952	28.095	*34.23*	*1.00*		
3,948^a^	−0.534	34.952	28.095	*34.46*	*1.14*		
328 (2 September 2007; −170.407°E, 87.827°N; depth: 3,990 m)							
50	−1.628	31.445	25.288	2.50	0.06	—	—
100^a^	−1.545	33.899	27.279	*3.36*	*0.06*	*0.20*	*0.01*
300	0.865	34.841	27.927	6.85	0.07	0.78	0.02
1,000	−0.218	34.896	28.034	12.40	0.12	1.31	0.02
1,001	−0.218	34.896	28.034	12.46	0.12	1.36	0.02
2,500^a^	−0.523	34.955	28.096	*29.08*	*0.20*	*3.62*	*0.04*
3,000	−0.534	34.955	28.097	23.06	0.17	3.48	0.04
3,750	−0.538	34.955	28.097	19.46	0.16	3.34	0.04
96 (September 11, 2015; −125.094°E, 88.3598°N; depth: 3,612 m)							
10	−1.505	28.344	22.769	2.60	0.14	0.24	0.13
50	−1.620	31.632	25.439	2.14	0.15	0.11	0.13
100	−1.453	33.776	27.177	2.54	0.15	0.13	0.13
200	0.189	34.623	27.792	4.61	0.18	0.35	0.13
300	0.801	34.812	27.907	6.91	0.17	0.25	0.13
1,000	−0.189	34.887	28.025	14.12	0.32	0.92	0.13
1,500	−0.437	34.926	28.069	16.32	0.24	2.03	0.13
2,000	−0.505	34.950	28.091	15.41	0.31	2.67	0.13
2,500	−0.516	34.956	28.097	16.14	0.25	—	—
3,000	−0.525	34.958	28.098	22.89	0.46	2.96	0.13
3,250	−0.529	34.958	28.099	23.71	0.42	2.88	0.13
3,495	−0.529	34.958	28.099	19.39	0.40	2.97	0.13
3,545	−0.529	34.958	28.099	25.29	0.66	2.64	0.13
101 (13 September 2015; 179.841°E, 87.497°N; depth: 3,996 m)							
23	−1.473	28.289	22.724	0.18	0.13	0.21	0.13
55	−1.310	31.559	25.373	1.50	0.17	0.10	0.13
99	−1.453	33.932	27.303	0.57	0.14	0.19	0.13
174	−0.092	34.530	27.732	4.04	0.27	0.31	0.13
259	0.781	34.810	27.907	4.27	0.19	0.46	0.13
499	0.556	34.867	27.967	5.61	0.20	0.64	0.13
999	−0.213	34.889	28.028	11.79	0.31	1.14	0.13
1,399	−0.412	34.921	28.064	17.24	0.35	2.18	0.13
1,899	−0.501	34.948	28.090	23.16	0.46	3.43	0.13
2,399	−0.517	34.955	28.096	19.96	0.43	3.68	0.13
2,899	−0.526	34.957	28.098	25.60	0.47	3.92	0.13
3,399	−0.532	34.957	28.098	28.47	0.63	4.03	0.13
3,881	−0.531	34.957	28.099	32.53	0.69	3.91	0.13
3,932	−0.531	34.957	28.099	26.58	0.54	3.80	0.13
134 (24 September 2015; 159.025°E, 84.844°N; depth: 3,170 m)							
300	1.010	34.855	27.928	3.90	0.17	0.61	0.13
1,000	‐0.236	34.891	28.031	9.79	0.27	1.18	0.13
2,000	‐0.504	34.946	28.088	17.74	0.32	2.94	0.13
2,500	‐0.511	34.952	28.094	16.89	0.47	3.19	0.13
3,000	‐0.526	34.955	28.096	20.64	0.61	3.48	0.13
3,000	‐0.526	34.955	28.096	21.18	0.49	3.59	0.13
3,040	‐0.527	34.955	28.096	21.50	0.54	3.63	0.13
3,090	‐0.528	34.955	28.096	20.98	0.49	3.71	0.13

a Only total concentrations were measured for these samples.

### 3.1 ^230^Th and ^231^Pa concentrations

^230^Th and ^231^Pa Concentrations were determined by isotopic dilution, after element separation and purification by chromatography. The analytical methodology followed for the samples collected in 2007 and 2015 in the MB and Alpha Ridge area is detailed in Valk et al. (2018). For the samples collected in 2005 in the MB and Mendeleev Ridge area and in 2007, 2009, 2011, and 2015 in the CB, ^230^Th and ^231^Pa were determined following the method described by Choi et al. (2001). For the 2015 samples the method was slightly modified as follows: (1) environmental grade acids were used to clean the sampling material and for the chemistry, to limit contamination for the determination of ε_Nd_ on the same samples; (2) the filtration was done through a single‐use 0.45 μm pore size filter cartridge (AquaPrep®; the 2011 samples remained unfiltered); (3) before analysis, the purified fractions of Th and Pa were both treated with 4 ml of concentrated perchloric acid to eliminate any organic component and then treated with concentrated HNO_3_ to remove the perchloric acid. The CB samples were measured at the University of British Columbia (Vancouver, Canada) on an inductively coupled plasma mass spectrometer (Element2, Thermo Scientific) coupled to a desolvating nebulizer system (Aridus II™, Teledyne CETAC) to increase the sensitivity. ^230^Th procedural blank contribution, mainly coming from the ^229^Th spike, was on average 14% for the 2009–2011 samples (10.21 ± 0.87 fg) and 2% for the 2015 samples (0.99 ± 0.09 fg). The Th recovery of the two chromatographic columns was estimated for the 2015 samples and found to be 77% in average. Isotope fractionation during ICPMS analysis was estimated at 0.6% per amu. As the U/Th chromatographic separation of the 2009 samples was done 8 years after the sample collection, measured ^230^Th_d_ concentrations were corrected from a substantial ^230^Th ingrowth. ^231^Pa procedural blank contribution, mainly coming from the ^233^Pa spike, was on average 56% for the 2009–2011 samples (46.5 ± 1.9 fg) and 70% for the 2015 samples (26.7 ± 1.6 fg). This large contribution resulted from over‐spiking but was well monitored by the set of chemical blanks and accurately corrected. The Pa recovery of the two chromatographic columns was estimated for the 2015 samples and found to be 80% in average.

### 3.2 Nd Isotopic Composition

The REE measurements came from the same initial natural samples used for Pa and Th. They were eluted with the Th fraction during the first chromatographic column and separated from Th on the second column in the 24 ml of 8 N HNO_3_. This fraction was dried and dissolved in 1.5 ml of HCl 1 N and was processed as described in Grenier et al. (2013). After the chemical extraction and purification of Nd, samples were dissolved in 2 μl of 2 N HCl, loaded on a rhenium filament and analyzed by thermal ionization mass spectrometry (TIMS) in static mode (ThermoFisher Scientific mass spectrometer Triton, Observatoire Midi‐Pyrénées, Toulouse). Thirteen analyses of Rennes standard and seven of La Jolla standard were performed to monitor instrumental drift and gave 0.511955 ± 0.000005 and 0.511854 ± 0.000009, respectively. All measurements were corrected for a machine bias of −0.000005, based on the accepted value being 0.511961 ± 0.000013 for Rennes (Chauvel & Blichert‐Toft, 2001) and 0.511858 ± 0.000007 for La Jolla (Lugmair et al., 1983).

## 4 Results

### 4.1 Hydrographic Change in the Atlantic and Bottom Waters

**Figure 5:**
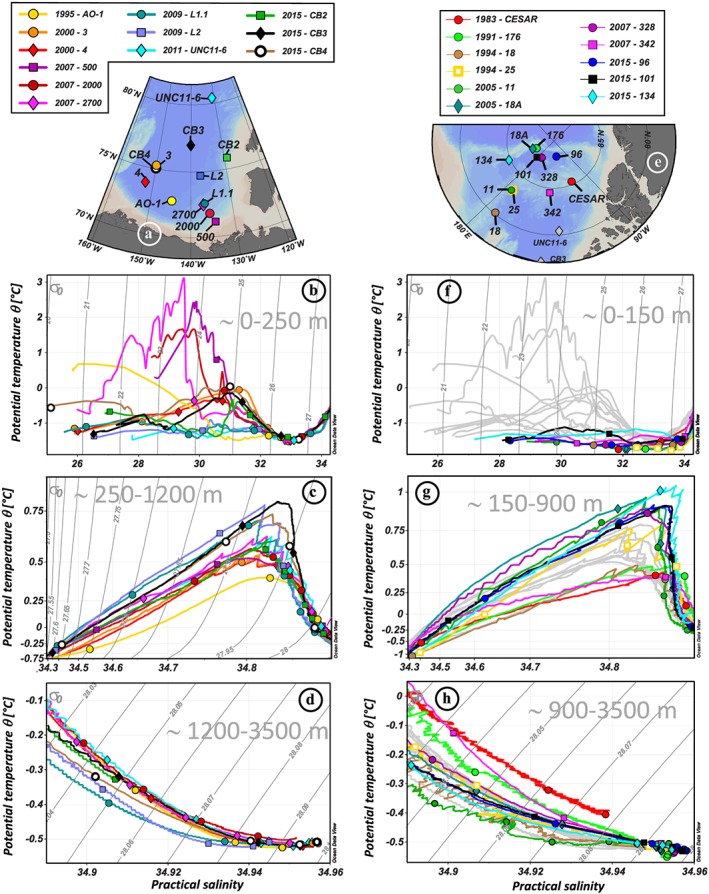
Potential temperature (in °C)—practical salinity (θ‐S) profiles of the Canada Basin stations (a–d) and of the Makarov Basin and Mendeleev/Alpha Ridge stations (e–h), showing the hydrological evolution of the shallow (b, f), intermediate (c, g), and deep and bottom (d, h) water masses between the ~1990s and ~2010s. Hydrological references are given in Figures 3 and 4. The symbols on the profiles represent the samples. Potential density contours σ_0_ are shown in solid grey. Canada Basin profiles are reported in light grey in the background of the Makarov Basin and ridges plots (e–h) to facilitate comparison.

Comparing temperature profiles taken in the same area at different time clearly shows that the Atlantic water has generally warmed in the Amerasian Basin between the ~1990s and 2010s. This warming was mainly observed in the upper part of the Atlantic water, around 300 m, for the MB and Mendeleev Ridge area, with a warming of ~0.4 °C between 1991 (station 176) and 2005 (station 18A) in the western MB and of ~0.1 °C between 1994 (station 25) and 2005 (station 11) on the Mendeleev Ridge (Figures 4a–4c). In contrast, on Alpha Ridge, the warming between 1983 (station CESAR) and 2007 (station 342) was not observed in the core of the Atlantic water layer (~450 m) but mainly between 500 and 1,500 m, in the lower part of the Atlantic layer and in the underlying uPDW. In the CB, the warming was centered in the upper part of the Atlantic water (Figures 3a–3e). An increase of temperature of ~0.1 °C was observed between 2007 (station 2700) and 2009 (station L1.1) between 300 and 500 m in the southern CB (Figure 5c). In the western CB, a deeper warming of ~0.25 °C was observed to a depth of 800 m between 2000 (station 3) and 2015 (station CB4). The warm Atlantic water observed over the Mendeleev Ridge in 1994, in the MB in 2005, and in the CB in 2007 and, to a greater extent, in 2009 documents the propagation of the awAW. The deep and bottom waters of the CB were fresher and lighter in 2009, compared to the other years sampled (stations L1.1 and L2; Figure 5d and Tables 1 and 2). In the MB, the deep waters were warmer, fresher, and lighter in the mid‐2000s than in 1991, and even slightly lighter in 2015, while the bottom MBDW were getting slightly saltier, warmer, and denser (Figure 5h and Table 2).

### 4.2 Geochemical Tracer Results

**Figure 6:**
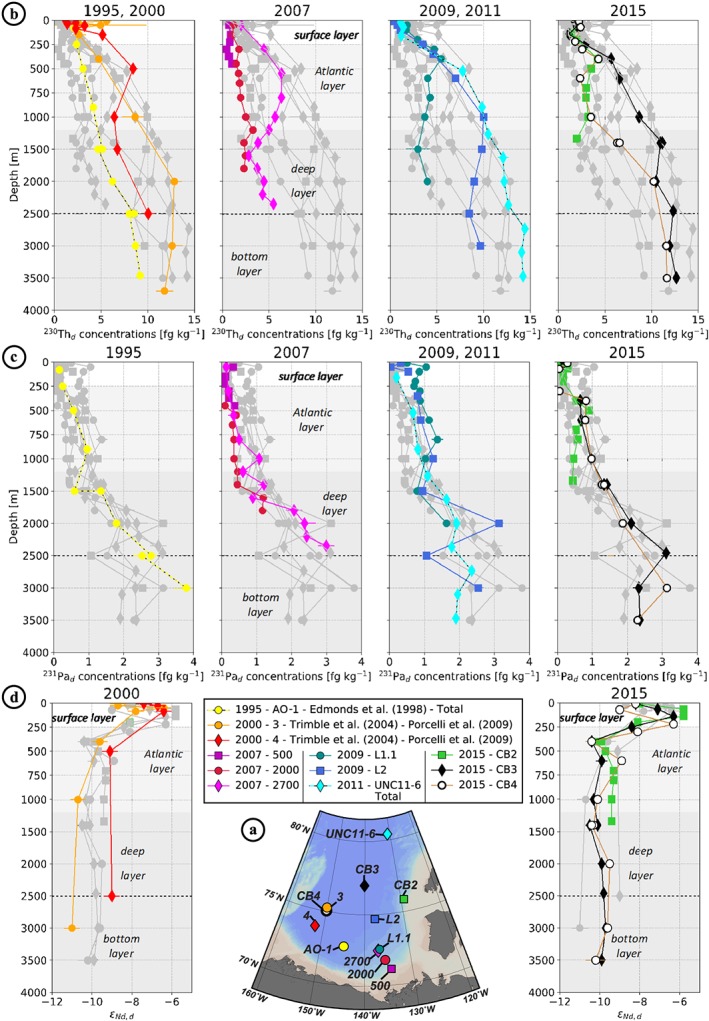
Vertical distribution of (b) ^230^Th_d_ concentrations (in fg kg^−1^), (c) ^231^Pa_d_ concentrations (in fg kg^−1^) and (d) ε_Nd_ of the Canada Basin stations (shown in a). The white, light grey, and dark grey backgrounds refer to the surface, intermediate Atlantic, deep uPDW and bottom CBDW layers, respectively, as subdivided in Figures 5b, 5c, and 5d. To facilitate the comparison between unfiltered and filtered profiles, dissolved profiles of ^230^Th and ^231^Pa were estimated at stations where samples were unfiltered, by multiplying the measured total concentrations by 0.8 for ^230^Th and by 0.95 for ^231^Pa (from the average dissolved/total ratios of the ^230^Th and ^231^Pa water column database http://climotope.earth.ox.ac.uk/data_compilations, by considering only Arctic stations, i.e., for station latitudes greater or equal to 75°N). These estimated dissolved profiles are represented by dashed lines.

**Figure 7:**
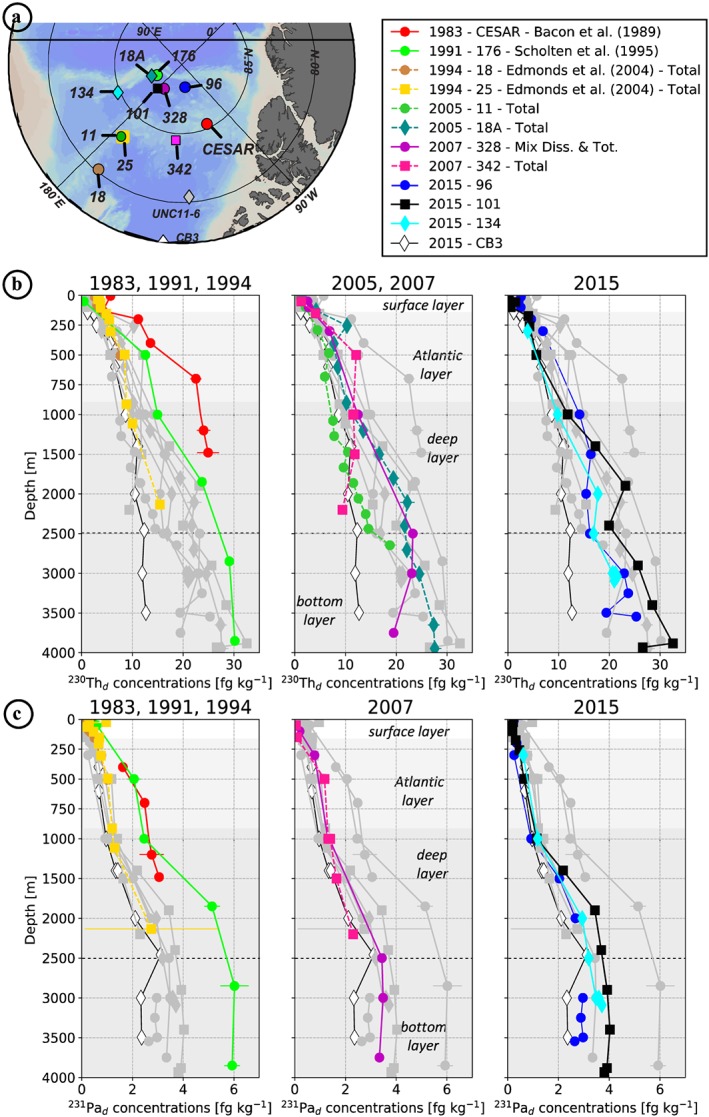
Vertical distribution of (b) ^230^Th_d_ concentrations (in fg kg^−1^) and (c) ^231^Pa_d_ concentrations (in fg kg^−1^) of the Makarov Basin and Mendeleev/Alpha Ridges stations (shown in a). The white, light grey, and dark grey backgrounds refer to the shallow, intermediate, and deep and bottom layers, respectively, as subdivided in Figures 5f, 5g, and 5 h. As in Figure 6, dissolved profiles of ^230^Th and ^231^Pa were estimated at stations where samples were unfiltered to facilitate the comparison between unfiltered and filtered profiles, by multiplying the measured total concentrations by 0.8 for ^230^Th and by 0.95 for ^231^Pa. These estimated dissolved profiles are represented by dashed lines.

**Figure 8:**
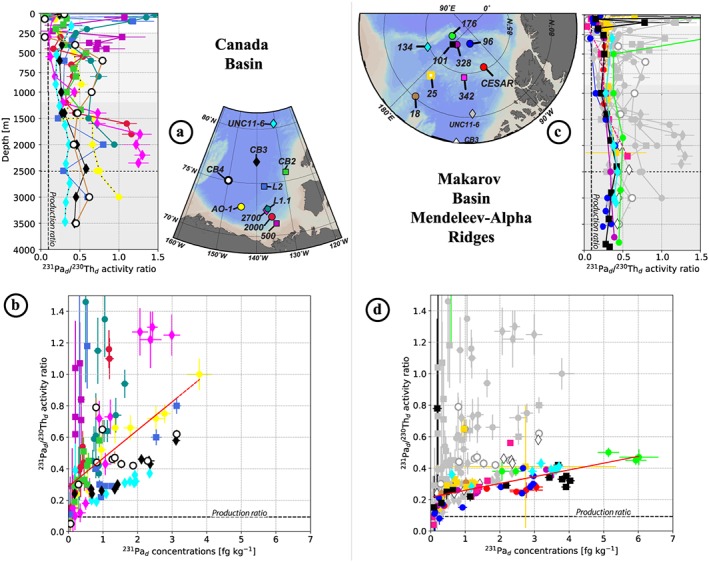
^231^Pa/^230^Th activity ratio as a function of depth (top) and of Pa concentrations (bottom; in fg kg^−1^) at the Canada Basin stations (a, b) and at the Makarov Basin and Mendeleev‐Alpha Ridge stations (c, d), in color, superimposed on the Canada Basin ones, in grey, for comparison. The dotted black line represents the production rate ratio (^231^Pa/^230^Th = 0.093; Anderson et al., 1983). The red line in (b) and (d) is the linear regression of the colored dots (excluding samples shallower than 50 m). In a steady state system, the slope of this red line reflects differences in the relative adsorption of Pa and Th. Steeper slopes reflect more intense scavenging of Th (higher *k*_*a*_) than for Pa. This is discussed further in the text.

^230^Th, ^231^Pa, and ε_Nd_ data from the new stations collected in the Amerasian Basin between 2005 and 2015 are compared to previously published samples collected between 1983 and 2000 (Figures 6 and 7). Most of the data refer to the dissolved fraction, from filtered samples, but some represent the total fraction, from unfiltered samples. We distinguished data referring to the total fraction by reporting them in italic in Table 2. To facilitate the comparison between unfiltered and filtered profiles, dissolved profiles of ^230^Th and ^231^Pa were estimated at stations where samples were unfiltered, by multiplying the measured total concentrations by 0.8 for ^230^Th and by 0.95 for ^231^Pa (from the average dissolved/total ratios of the ^230^Th and ^231^Pa water column database http://climotope.earth.ox.ac.uk/data_compilations, by considering only Arctic stations, i.e., for station latitudes greater or equal to 75°N). These estimated dissolved profiles are represented by dashed lines in Figures 6 and 7. In order to track fractionation between ^230^Th and ^231^Pa, dissolved ^231^Pa/^230^Th activity ratios are also shown, for each area, in Figure 8.

The geochemical tracer results reported from the different areas of the Amerasian Basin compare well with the published data (Figures 6 and 7). Concentrations of ^230^Th and ^231^Pa are consistently higher in the MB and over ridges than in the CB. Profiles from the deep central CB and MB show smaller increase with depth in the bottom layer, reflecting particulate scavenging by sediment resuspension (Anderson et al., 1983; Rempfer et al., 2017; Scholten et al., 1995). ^230^Th exhibits a greater spatial heterogeneity than ^231^Pa, also consistent with the previously published profiles. Lower ^230^Th concentrations are found in the sampling sites close to the boundary circulation, while higher concentrations characterize the central basins, consistent with the gradient described in Figure 2. New 2015 ε_Nd_ profiles fall well within the range defined by the profiles collected in the western CB in 2000 (Porcelli et al., 2009).

## 5 Discussion

### 5.1 Spatial and Temporal Variability in ^230^Th and ^231^Pa Concentrations and Seawater ε_Nd_

Overall, none of the ^230^Th profiles collected after 2000 exceeds the concentration levels of the published, pre‐2000s, deep profiles; they are equally high or significantly lower. This observation suggests that the intensity of particulate scavenging in the Amerasian Basin has, in the last two decades, been maintained or increased depending on the area, preventing ^230^Th concentrations from building up due to uranium (U) decay.

#### 5.1.1 Variability of ^230^Th and ^231^Pa in the CB

In the interior of CB, the ^230^Th profiles measured at stations 3 (orange circles) sampled in 2000 (Trimble et al., 2004), UNC11‐6 (cyan diamonds) sampled in 2011, and CB3 (black diamonds) sampled in 2015 (Figure 6a) are very similar (Figure 6b), suggesting that the balance between^230^Th removal by particle scavenging and production from U decay was maintained over that time period. By contrast, at the southern CB coastal stations 2000 (dark red circles) and 500 (purple squares) sampled in 2007, ^230^Th concentrations are significantly lower compared to the 1995 profile measured at AO‐1 in 1995 (yellow circles; Edmonds et al., 1998). A similar change is observed for ^231^Pa between station 2000 (dark red circles) and AO‐1 (yellow circles; Figure 6c). These observations indicate increasing particle scavenging in the southern coastal region of the CB between 1995 and 2007. In the transition zone between the southern margin and the interior zone discussed above, station 4 (red diamonds) sampled in 2000 (Trimble et al., 2004), 2700 (pink diamonds) sampled in 2007, and L2 (blue square) sampled in 2009 exhibit a pronounced ^230^Th maximum in the Atlantic layer, suggesting a lateral exchange signature, as schematized in Figure 2b. Particularly remarkable is the sharp difference in the profiles measured in 2007 at stations 2000 and 2700 (Figure 6b), as these two stations are only 51 nautical miles apart and were sampled within a few days (see sampling dates and locations in Table 2).

#### 5.1.2 Variability of ε_Nd_ in the CB

Differences between coastal and interior regions of the CB are also observed in ε_Nd_ profiles (Figure 6d). Stations sampled in 2000 (Porcelli et al., 2009) exhibited significant differences in ε_Nd_, with station 4 (red diamonds), identified as a transition zone station from its ^230^Th profile (Figure 6b), having significantly more radiogenic values than station 3 (interior basin). This observation suggests that in 2000, these two locations were in areas of distinct circulation, with little lateral exchange between them. The southern, more radiogenic 2000 profile resembles the one observed at CB2 in 2015, on the eastern CB margin (red diamond and green square in Figure 6d): they are likely representative of the water masses associated with the boundary circulation. The northern, less radiogenic 2000 profile (station 3, orange circles) resembles those observed at the deep stations in 2015 (black diamond and white circle profiles): these are likely representative of the central basin water masses. The more pronounced horizontal ε_Nd_ gradient between the boundary versus central CB in 2000 compared to 2015 reflects increasing lateral exchanges between these two areas between 2000 and 2015.

#### 5.1.3 Contrasting ^230^Th, ^231^Pa, and ε_Nd_ Variability in the 2015 CB

The two 2015 deep stations (CB3 and CB4; Figure 6a) exhibit very similar profiles in ε_Nd_ (Figure 6d) and ^231^Pa (Figure 6c), but not in ^230^Th (Figure 6b). For the latter, the Atlantic layer at CB4 (white circles) exhibits much lower concentrations than at CB3 (black diamonds), resulting in higher ^231^Pa/^230^Th ratios (Figures 8a–8b). These differences reveal ongoing processes for which ^230^Th is more sensitive than ^231^Pa and ε_Nd_, most likely an increase in particle fluxes that removes ^230^Th faster than the two other geochemical tracers, which are less susceptible to scavenging. CB4 is in closer vicinity to the boundary circulation and exhibits ^230^Th concentrations in the Atlantic water similar to those found at the 2015 coastal station CB2 (green squares). However, unlike CB4, CB3 also has lower ^231^Pa concentrations and more radiogenic ε_Nd_. These results suggest that in 2015, the coastal region of the CB is impacted by higher fluxes of particles, which started to affect CB4. It seems to also be the case in 2009 at station L1.1 (blue circles), which is very close to station 2700 sampled in 2007 (pink diamonds). The ^230^Th profile at station L1.1 resembles more that at CB2 than at 2700, while ^231^Pa profiles measured in 2009 at L1.1 are closer to 2700 measured in 2009 than CB2 measured in 2015.

To summarize, in the CB, the comparison of published and new geochemical profiles suggests (1) enhanced fluxes of particles along the radiogenic boundary circulation pathway after 2000, evidenced by lower ^230^Th and ^231^Pa concentrations and ^230^Th‐^231^Pa fractionation; (2) a transition zone between the margin and the CB interior where lateral exchanges of water was between the margin and the basin interior was captured in 2000, 2007, and 2009 by nonlinear ^230^Th profiles, showing a concentration maximum in the Atlantic layer; (3) an area north of 75°N in the central basin subject to low exchanges with the boundary circulation, with more negative ε_Nd_ and a particle flux maintained at relatively low level.

#### 5.1.4 Variability of ^230^Th and ^231^Pa in the MB

In the Makarov Basin and adjacent ridges area (hereafter MBAR), the highest dissolved ^230^Th and ^231^Pa concentrations were measured over Alpha Ridge in 1983 (red circles; Bacon et al., 1989) and the northern MB in 1991 (green circles; Scholten et al., 1995; Figure 7). Building up such high concentration requires a residence time exceeding several decades in a region subjected to a very low particle flux region, as would be expected under permanent sea ice cover, to allow ingrowth of the two nuclides from their parent U isotopes. Dissolved ^231^Pa/^230^Th ratios at these two stations are not as high as generally observed in the CB (Figures 8c–8d), which is also consistent with a multidecadal residence time under permanent sea ice. As a water mass enters a region subjected to a lower particle flux, its ^230^Th concentration build up faster than ^231^Pa concentration because of the differences in production rate and residence time with respect to scavenging between the two radionuclides, resulting in a gradually decreasing dissolved ^231^Pa/^230^Th ratio with time. However, the MB profile comparison highlights (1) a significant decrease of ^231^Pa in the post‐2000 profiles, not as marked in ^230^Th (Figure 7c), and (2) an inflexion in ^230^Th profiles with a minimum around 2,500 m for all the 2015 profiles (blue and black profiles in Figure 7a). The comparison of the two Alpha Ridge profiles shows significantly lower concentrations of ^231^Pa at the 2007 station, even more pronounced for ^230^Th, surprisingly tending toward CB concentrations with depth (pink square profile in Figure 7). In the following, we further develop the hypothesis presented above for the CB and hypotheses to explain the MBAR features.

### 5.2 Increased Particle Flux in the Margin Area

**Figure 9:**
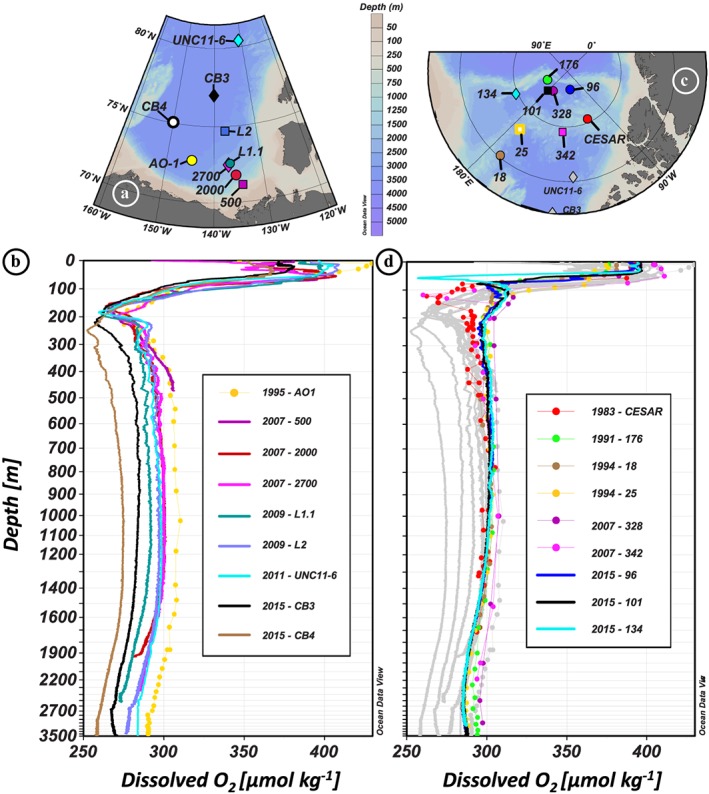
Dissolved oxygen concentrations (in μmol kg^−1^) measured at the different stations shown in (a) and (c), for (b) the Canada Basin and for (d) the Makarov Basin and Mendeleev‐Alpha Ridges, in color, superimposed on the Canada Basin ones, in grey, for comparison. References are given in Figures 3 and 4.

In the CB, the low ^230^Th and ^231^Pa profiles of the two 2007 coastal stations compared to the coastal 1995 profile reveal the impact of an enhanced vertical particle flux. The 2007 stations were located on the margin close to the Mackenzie River mouth, an area more and more seasonally free of ice (Figure S1): particulate riverine material and biological productivity could both explain an enhanced vertical particle flux at these coastal stations. The deeper 2007 coastal stations (stations 2000 and 2700) exhibit lower dissolved oxygen concentrations than measured in 1995 at station AO‐1, supporting an increasing flux of biogenic particles (Figures 9a–9b). Ice rafted particles may also be released during the seasonal ice melt and possibly reinforce the particle flux and scavenging rate in this margin area (Baskaran et al., 2003; Trimble et al., 2004).

**Figure 10:**
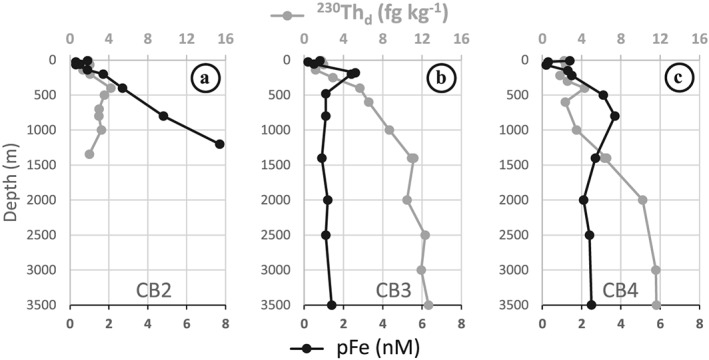
Vertical distribution of ^230^Th_d_ concentrations (in fg kg^−1^; grey curves) and particulate Fe concentrations (pFe, in nM; black lines, from Li, 2017) in the Canada Basin in 2015, at (a) station CB2 (eastern CB), (b) station CB3 (northern CB), and (c) station CB4 (western CB). ^230^Th and pFe ranges are kept the same for the three stations to highlight differences.

In addition to enhanced vertical fluxes of particles in the margin area by increased biological productivity, our 2015 results also suggest lateral fluxes of continental particles. As noted above, the 2015 profiles measured at the two deep stations CB3 and CB4 have similar ε_Nd_ (Figure 6d) and ^231^Pa (Figure 6c) concentrations in the Atlantic layer, but lower ^230^Th concentration at CB4 (Figure 6b), while at the coastal station (CB2), ε_Nd_ was more radiogenic and both ^230^Th and ^231^Pa concentrations were lower than at CB3. Comparing the ^230^Th profiles to particulate iron (pFe) measured at the same stations (Figure 10; Li, 2017) reveals that the ^230^Th minimum observed in the Atlantic layer at CB4 coincides with a maximum of pFe, both of which are not observed at CB3 (Figures 10b–10c). These observations suggest scavenging by lithogenic particle at CB4, possibly coming from the margin or the Northwind Ridge (this ridge rises to ~520 m below the surface, 250 km west of CB4; see Figure 3a). Moreover, the Atlantic layer at the coastal station CB2 exhibits much higher pFe and much lower ^230^Th concentrations than at the deep stations (Figure 10a), suggesting higher flux of coastal particles that scavenges both ^230^Th and ^231^Pa. The more radiogenic ε_Nd_ below 500 m at CB2 compared to CB3 and CB4 (Figure 6d) further suggests that this signature is imparted from margin sediments, which is consistent with the ε_Nd_ of the acetic acid leachates of surface sediments measured in the area (Haley & Polyak, 2013). The suggested impact of coastal particles on ^230^Th, ^231^Pa, and ε_Nd_ data at CB4 cannot only be the result of coastal processes transmitted offshore by advection. Instead, coastal particles must be advected from the margin to CB4 to explain the pFe data. The transport of dissolved and particulate lithogenic signature from nepheloid layers formed by sediment resuspension along the CB margin across the area of boundary circulation could explain the distribution of all these four tracers. The occurrence of nepheloid layers has indeed been reported in the CB margin area (e.g., Ehn et al., 2019; O'Brien et al., 2006, 2013). Coastal water and particles could also be transported through eddies generated in the southern CB boundary current (Watanabe, 2011; Zhao et al., 2014), whose number has intensified over the last two decades (Zhao et al., 2016).

The high dissolved ^231^Pa/^230^Th activity ratios in the Atlantic layer of most CB coastal stations collected after 2000 (Figures 8a–8b) are consistent with the preferential scavenging of ^230^Th by lithogenic particles (Chase et al., 2002). Such high ^231^Pa/^230^Th activity ratios are not observed in the MBAR area (Figures 8c–8d) where dissolved oxygen concentration profiles are not decreasing with time as in the CB (Figures 9c–9d), suggesting that fluxes of lithogenic and biogenic particles are lower and more constant in time in the MBAR area. This difference is consistent with the evolution of sea ice coverage, more persistent in the MBAR area than in the CB (Figure S1), limiting biological productivity and ice rafted debris release.

### 5.3 Spatial Characterization of an Area of Lateral Exchanges Between the Boundary Circulation and Northern Central CB

The nonlinear profiles observed in 2000 (station 4), 2007 (station 2700), and 2009 (station L2; Figure 6b) document the presence of an area where coastal low‐^230^Th and offshore high‐^230^Th waters influence the ^230^Th profiles to different extents at different depths. At these three stations, the relatively high concentrations measured at intermediate depths reflect advection or mixing of water originating from the northern CB. The southern limit of this mixing area in 2007 is fairly well captured by the sharp difference in ^230^Th concentrations and potential temperature within the Atlantic layer between the most offshore station 2700 and the more inshore station 2000 located 51 nautical miles south. At station 2700, the Atlantic water has higher ^230^Th concentrations (Figure 6b), a higher temperature maximum (Figure 3c; inset), and a temperature profile showing zigzags indicating weak turbulence and double‐diffusive intrusions (Woodgate et al., 2007)—consistent with an offshore northern CB origin (McLaughlin et al., 2009). In contrast, the low ^230^Th concentration and smooth θ‐S profile in the Atlantic layer of station 2000 is consistent with a boundary origin (Figures 5c and 6b). These two profiles are likely located across the front separating the cyclonic boundary current from the central anticyclonic flow (McLaughlin et al., 2009). The 1995 ^230^Th profile did not exhibit such deviations from linearity and was therefore likely located south of this front.

**Figure 11:**
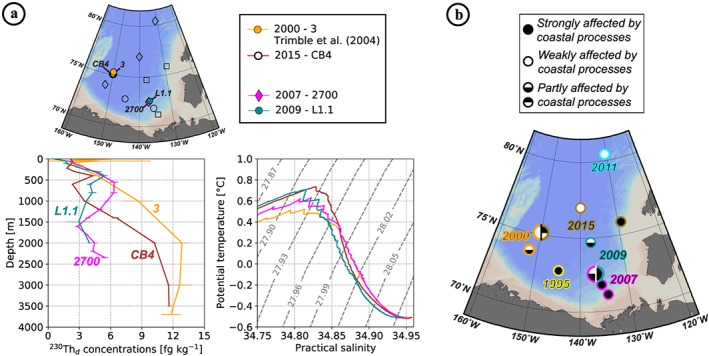
Comparison of ^230^Th_d_ concentrations (in fg kg^−1^) and θ‐S profiles (with superimposed isopycnal σ_0_) at stations of very close location but of different sampling year. (b) Schematic representation of the Canada Basin (CB) areas as defined from the ^230^Th profile features. Each station location is characterized following its degree of impact by coastal processes: weak, strong, or intermediate (white, black, and black/white filled circles, respectively), closely related to the extension of the boundary, northern, and mixed water areas. Color contours refer to the year of station sampling (same color code as in Figure 1). Larger circles, cut in two halves, represent temporal variability of the spatial extension of boundary processes from the stations visited twice (a).

Interestingly, it seems that the location of this front has moved further north between 2007 and 2009. Station L1.1, sampled in 2009 (blue circles), is almost at the same location as station 2700 sampled in 2007 (most offshore station; pink vs. green profiles in Figure 11a). It is difficult to explain the sharp decrease observed in ^230^Th concentrations within the Atlantic layer between 2007 and 2009 only as a result of increased particle flux and scavenging, considering the residence time of Th at these depths. Instead, it most likely reflects a greater relative proportion of “margin” versus “offshore” water at this location in 2009. The smoother θ‐S within the Atlantic layer and more radiogenic, ^230^Th‐depleted signal found at 1,000 m at the 2015 station CB4 (white circles, Figure 6), compared to the nearby 2000 station 3 (orange circles; Figure 6) sampled 15 years before may also reflect the northward displacement of this front and increase lateral exchanges between the margin area and the central CB during the last two decades (orange vs. brown profiles in Figures 11a and 6d). Figure 11b summarizes, through the characteristics of the ^230^Th vertical profiles, the spatial distribution and temporal variability of the impact of coastal processes and lateral exchanges.

The lateral exchange variability suggested in the CB may be related to the propagation in the Arctic of the awAW: associated with a larger volume of inflowing Atlantic water (Karcher et al., 2003; Schauer et al., 2004), this awAW propagated as a stronger intermediate flow, which presumably reinforced the two circulation schemes of the CB (anticyclonic circulation in the deep basin; Coachman & Barnes, 1963; Newton & Coachman, 1974; boundary cyclonic circulation) and enhanced the lateral mixing between the cyclonic boundary circulation and the anticyclonic central circulation. This is supported by the coexistence of the temporal increase of vertical variability within the ^230^Th profiles with the temporal increase of temperature of the upper Atlantic layer (Figure 11a). The eddy activity and its intensification over the last decades (Timmermans et al., 2008; Watanabe, 2011; Zhao et al., 2016) could also be partly responsible for the lateral exchange variability suggested by our profiles.

### 5.4 Lateral Exchanges in Other Areas of the Amerasian Basin

#### 5.4.1 Lateral Exchange in the Atlantic/Deep Layer Between the Northern CB and the Southern Alpha Ridge

**Figure 12:**
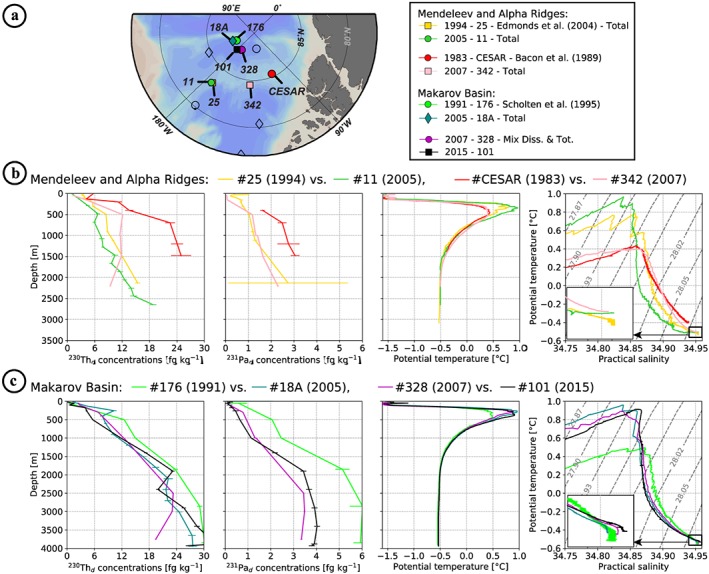
Comparison of geochemical (^230^Th_d_ and ^231^Pa_d_ concentrations, as shown in Figures 5 and 6, in fg kg^−1^) and hydrological (potential temperature θ vertical profiles, in °C, and θ‐S profiles with superimposed isopycnal σ_0_) properties at stations of very close location but of different sampling year (also referred in the text as “twice visited,” except for the Alpha Ridge stations that are too far apart to be considered as such). Concerned stations are identified in the map shown in (a) and properties are shown in (b) for the Mendeleev and Alpha Ridge stations and in (c) for the Makarov Basin ones. The θ‐S plot insets show a zoom on the θ‐S bottom water characteristics delimited by the black squares.

Unlike at the stations in the CB, MB and Mendeleev Ridge areas, waters sampled in the Alpha Ridge (AR) area (station CESAR and 342) before and after 2000 do not exhibit significant hydrological changes (Figures 12b). The awAW propagation, seen in other regions, was not identifiable at station 342 in 2007, while it was evident at station 328 sampled the same year in the MB (Figure 12). This suggests the relative isolation of the water column in AR area from the MB and CB circulation. In addition, the high ^230^Th_d_ and ^231^Pa_d_ concentrations measured in 1983 in the northern AR (station CESAR) reveal the long isolation of the water column in this area under low flux of particles, allowing ingrowth of the two radionuclides (see Figure 2c; Bacon et al., 1989). In 1996, Smethie et al. (2000) consistently found low CFC‐11 concentrations and ^3^H‐^3^He ages of 25 years on average below 300 m in this area.

Yet much lower ^230^Th_d_ and ^231^Pa_d_ concentrations were measured at station 342 in 2007, with a surprising decrease in ^230^Th_d_ with depth (Figure 12b). Such differences between the 1983 and the 2007 profiles imply the presence of intermediate/deep waters at the 2007 station distinct from the water mass sampled in 1983. From the comparison of the hydrological profiles at these two AR stations to those of the whole station set reported here (Figures 3, 4, 5 and 9), AR waters appear to be dominantly derived from CB waters and, more particularly, from colder, saltier, and denser waters predating the awAW propagation in the CB. The largest hydrological differences observed between the AR stations collected in 1983 and 2007 are actually not in the AW core (~500 m) but in the lower halocline and lower AW, where the 2007 station is warmer (Figure 12b). These geochemical and hydrological features of the two AR stations are consistent with the lower AW and uPDW in 2007 being relatively recently renewed by CB waters. The ^230^Th_d_ and ^231^Pa_d_ profiles are consistent with this hypothesis, as well as the dissolved oxygen (Figures 7 and 9d). The water renewal had to occur about 10 years before the sampling of the 2007 station, that is, predating the awAW arrival in the CB (i.e., 2003; Woodgate et al., 2007) but postdating the low CFC, ^3^H and ^3^He observed in the area in 1996 (Smethie et al., 2000). It is possible that the ^230^Th_d_ content of the uPDW in this area was additionally impacted by scavenging. Indeed, the 2007 station was collected on the deep southwestern flank of the AR, in contrast to the 1983 station which was situated over the top of the AR (Figures 12a). Sediment resuspension from the Alpha Ridge slope that rises to 1,600 m, 60 km further north (84.9°N, 134.3°W; http://www.gmrt.org) and to 1,300 m, 140 km further northeast of the 2007 station (85°N, 126°W) could impact the area sampled in 2007.

#### 5.4.2 Lateral Exchanges Between the Deep Northern CB and the Deep MB

Intrusions of CB waters into the MB above the deeper sill between the Mendeleev and Alpha Ridges (~2,400 m) could explain the ^230^Th_d_ concentration minimum observed within the Deep Transitional Layer (DTL, 2,000–2,500 m; Timmermans et al., 2003) in 2015 in the MB (stations 96, 101, and 134; Figures 7 and 12c). Indeed, the deep and bottom layers of the CB are characterized by significantly lower concentrations of ^230^Th than the MB; the uniformity in the ^231^Pa concentrations of the MB and northern CB at this depth also support such exchanges. In addition, the MB ^231^Pa/^230^Th activity ratios show a consistent deviation around this depth toward slightly higher, CB‐like, values (Figure 8c). The fact that this ^230^Th minimum is clearly observed in the eastern MB (station 96) further suggests that this CB signature spreads through the entire Basin (i.e., east of 180°). Hydrological features such as the θ‐S convexity and O_2_ minimum observed within the DTL at the MB stations also support exchanges of CB deep waters toward the MB (Figures 4, 5h, and 9d). Although the direction of exchanges we suggest here conflicts with former hydrological study conclusions that invoked a dominant flux of DTL water above the Mendeleev/Alpha Ridge from the MB toward the CB (e.g., Carmack et al., 2012; Rudels, 2009), Swift et al. (1997) also invoked a flow from the CB toward the MB above the Mendeleev Ridge to explain the local silicate maximum observed in the MB around 2,400 m depth.

Intrusions of CB deep waters into the MB could also explain the warmer and saltier water observed in the bottom waters at the MB and Mendeleev Ridge stations after 2000 (insets in the θ‐S plots in Figures 12b–12c). Nonetheless, considering the gradual decrease of tracer—especially ^231^Pa_d_—concentrations with depth below 3,000 m, coupled to a gradual increase of dissolved O_2_ (Figures 9b and 12c), we agree with Middag et al. (2009) and Roeske et al. (2012) that MB bottom water is also made of some colder and fresher Amundsen Basin Deep Water (ABDW) that likely came over the Lomonosov Ridge (between 2,000 and 2,500 m) and sank due to its higher density than the local bottom waters (Timmermans et al., 2005).

#### 5.4.3 Lateral Exchanges Between the Eurasian Basin and the Central Makarov Basin

As previously mentioned, the data reported in this work suggest that fluxes of lithogenic and biogenic particles have been weaker and more constant in time in the MBAR area than in the CB (see section 5.2). Yet significant differences are observed between the ^230^Th_d_ and ^231^Pa_d_ profiles in the MB before 2000 (Station 176; Scholten et al., 1995) and those at the stations visited after 2000 (Figure 12c): post‐2000s profiles exhibit lower radionuclide concentrations, especially pronounced in ^231^Pa_d_, compared to the 1991 profile. The high ^230^Th_d_ and ^231^Pa_d_ concentrations measured in 1991 in the northern MB reflect, as for the 1983 station collected over the AR, a long isolation of the area from the dynamic boundary circulation of the MB and a low flux of particles (Scholten et al., 1995). The difference of ^231^Pa_d_ concentration between this 1991 station and those visited after 2000 increases with depth. It is difficult to explain the pre‐ versus post‐2000s radionuclide differences observed in the MB as a stronger flux of particles, as a change in particle flux strong enough to explain the ^231^Pa changes in depth should have also led to a much larger ^230^Th depletion than those observed (Figure 12c). Not to mention that, despite the evolution of sea ice coverage since the 1990s, most of the MB has remained permanently covered by sea ice over these years (Figure S1), limiting an enhancement of productivity.

Therefore, our geochemical results suggest that waters with lower ^230^Th concentrations and lower ^231^Pa/^230^Th activity ratios (due to lower concentrations of ^231^Pa relatively to ^230^Th) propagated into the MB after 1991. CB waters are characterized by too high ^231^Pa/^230^Th activity ratios and too low oxygen concentrations to represent a valid candidate. In contrast, consistent lower ^230^Th concentrations and lower ^231^Pa/^230^Th activity ratios have been documented in the Eurasian waters (Edmonds et al., 2004), as well as similar oxygen concentrations (Schauer et al., 2002). The presence of the awAW at the stations visited in the MB after 2000 supports the occurrence of exchanges between this previously isolated area and the topographically steered intermediate circulation (Figure 12c).

## 6 Conclusions

**Figure 13:**
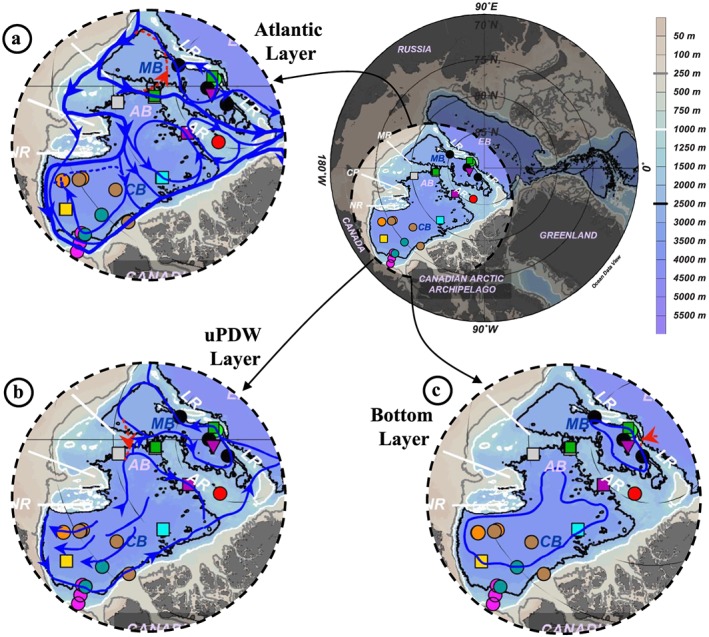
Schematic representations of Amerasian Basin circulation layers: (a) Atlantic water layer (~250–1,000 m); (b) uPDW layer (~1,000–2,500 m); (c) bottom layer (~2,500 m bottom; flow direction not given, due to uncertainties). The 250, 1,000, and 2,500 m isobaths are represented by grey, white, and black lines, respectively. EB = Eurasian Basin; AB = Amerasian Basin; MB = Makarov Basin; CB = Canada Basin; LR = Lomonosov Ridge; AR = Alpha Ridge; MR = Mendeleev Ridge; NR = Northwind Ridge; CP = Chukchi Plateau. Dotted red pathways—within the MB in (a), over the MR toward the CB in (b), and over the LR toward the MB in (c)—are paths that were likely dominant in the past but are likely minor nowadays.

This work presents a compilation of published and new depth profiles of ^230^Th, ^231^Pa, and ε_Nd_ collected between 1983 and 2015 in the Amerasian Basin of the Arctic Ocean, in particular in the Canada and Makarov basins and over the Mendeleev and Alpha Ridges. The distribution of these geochemical tracers allow an assessment of the spatial and temporal variability in particle flux and water mass circulation and mixing in these different areas. A representation of intermediate‐to‐bottom layer circulation, including pathways likely dominant in the past but suggested to be more minor recently (section 5.4) as well as the circulation from the main conclusions (summarized below) is outlined in Figure 13.

A temporal decrease in concentrations of the particle‐reactive tracers ^230^Th and ^231^Pa is observed in the whole Amerasian Basin and results from particle scavenging, and mixing and circulation variability. Particularly in the Canada Basin, the intensification of lithogenic and biologic particle fluxes likely results from margin sediment transport and enhanced biological production, respectively, in relation to sea ice retreat.

Imprints of increased lateral exchange are also reported in several areas of the Amerasian Basin. In the Canada Basin, they are represented by the coexistence of high ^230^Th Atlantic water of northern origin overlying low ^230^Th waters that have their origin in the boundary; the spatial extent of this area of lateral exchange seems to vary in time. The geochemical and hydrological characteristics at the southern flank of the Alpha Ridge in 2007 reflect the occurrence of lateral exchange in the Atlantic layer and uPDW with Canada Basin waters in the early 2000s, in contrast with the isolated character of this area reported in the 1980–1990s. Similarly, the geochemical and hydrological characteristics reported in the MB suggest that lateral exchange has connected the previously isolated northern MB with the main circulation (the pre‐2000s Makarov Basin circulation was likely restricted to the red arrow shown in Figure 13a; blue arrows show the connection of the offshore Makarov Basin with the boundary circulation suggested from the post‐2000s profiles). The results reported in this study also suggest increased intrusion of Canada Basin deep waters over the Mendeleev‐Alpha Ridge sill into the Makarov Basin, impacting the Deep Transitional Layer (2,000–2,500 m) waters of the Makarov Basin (while a dominant overflow from the Makarov Basin toward the Canada Basin was previously suggested, as represented by the red arrow in Figure 13b). Canada Basin Deep Water intrusion also seems to mix with deeper waters of the Makarov Basin and decrease the Amundsen Basin Deep Water (ABDW) signature of the Makarov Basin Bottom Water (ABDW overflow represented by the red arrow in Figure 13c).

These conclusions are based on sparse samples, in space and time. Additional sampling is needed to refine the hypotheses proposed in this study. There is a need to better document the coastal Makarov Basin, from the Lomonosov Ridge to the Mendeleev Ridge, and the northern Canada Basin, from the Chukchi Plateau to the northeast Canadian Arctic Archipelago (CAA). It would be of interest to sample the Alpha Ridge again over the southern and northern flanks to determine whether this area is isolated, or if changes in the Arctic circulation have led to exchange of waters between this area and the Canada Basin. Finally, with the circulation changes suggested in the Makarov Basin, it would also be interesting to monitor the water circulation in the vicinity of the western Lomonosov Ridge, by sampling the Greenland side of the Makarov Basin, Alpha Ridge and Amundsen Basin, to better document the composition of waters flowing through Nares Strait (between the CAA and Greenland) and those returning to Fram Strait. The circulation and particle distribution of the Amerasian Basin are sufficiently heterogeneous to make these geochemical tracers a useful tool to study the Arctic Ocean state and evolution, though the utility of these tracers may be challenged if mixing and particle concentration dramatically increase and eventually erases the current gradients.

## Supporting information

Supporting Information S1
