# Supplementary material for: Changes in Circulation and Particle Scavenging in the Amerasian Basin of the Arctic Ocean over the Last Three Decades Inferred from the Water Column Distribution of Geochemical Tracers
Source: J Geophys Res Oceans. 2019 Dec 18;124(12):9338–63. doi: 10.1029/2019JC015265 (PMC7006760; doi:10.1029/2019JC015265)
Supplement: Supplementary file 1 — Supporting Information S1 [file JGRC-124-9338-s001.docx]

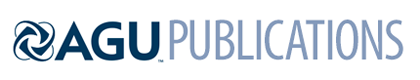


*Journal of Geophysical Research - Oceans*

Supporting Information for

**Changes of circulation and particle scavenging in the Amerasian Basin of the Arctic Ocean inferred from the distribution of radioactive and radiogenic tracers in the last three decades**

Melanie Grenier^1^, Roger François^1^, Maureen Soon^1^, Michiel Rutgers van der Loeff^2^, Xiaoxin Yu^1^, Ole Valk^2^, Christelle Not^3^, S. Bradley Moran^4^, R. Lawrence Edwards^5^, Yanbin Lu^5^, Kate Lepore^6^, and Susan E. Allen^1^

^1^Department of Earth, Ocean and Atmospheric Sciences, University of British Columbia, 2207 Main Mall, Vancouver, BC V6T 1Z4, Canada.

^2^Alfred Wegener Institute, Helmholtz Centre for Polar and Marine Research, Am Handelshafen 12, 27570 Bremerhaven, Germany.

^3^Department of Earth Sciences, The University of Hong Kong, Hong Kong SAR.

^4^College of Fisheries and Ocean Sciences, University of Alaska Fairbanks, Fairbanks, AK 99775, USA.

^5^Department of Earth Sciences, University of Minnesota, 310 Pillsbury Dr SE, Minneapolis, MN 55455, USA.

^6^Mount Holyoke College, South Hadley, MA 01075, USA.

**Content of this file**

Figure S1

**Introduction**

This supporting information provides the September Arctic sea ice extension and concentration from the National Snow & Ice Data Center for the years corresponding to the stations reported in this study.

**Figure S1.** September Arctic sea ice extension and concentration from the National Snow & Ice Data Center (https://nsidc.org/data/seaice_index/archives; ftp://sidads.colorado.edu/DATASETS/NOAA/G02135/north/monthly/images/09_Sep/) for the years corresponding to the stations reported in this study. The station locations are reported to show the ice coverage characteristics for each station (red dots).
